# Evolving Epidemiology of Pediatric Respiratory Syncytial Virus (RSV) Cases Around COVID-19 Pandemic: Impact and Clinical Insights, Retrospective Cohort Study

**DOI:** 10.1007/s44197-024-00218-4

**Published:** 2024-04-04

**Authors:** Deema Gashgarey, Mohammed Alsuhaibani, Raghad Alhuthil, Hattan Alhabshan, Azzam Alabdulqader, Rakan Badran, Abdulaziz Balhmar, Haifa Aldawood, Esam A. AlBanyan, Salem AlGhamdi, Suliman AlJumaah, Ohoud AlYabes, Sami Al-Hajjar

**Affiliations:** 1 Pediatrics Department, King Faisal Specialist Hospital & Research Centre (KFSHRC), Riyadh, Saudi Arabia; 2 College of Medicine, Alfaisal University, Riyadh, Saudi Arabia

**Keywords:** Respiratory syncytial virus, RSV, COVID-19, Pediatric, Epidemiology

## Abstract

**Background:**

The burden of respiratory syncytial virus (RSV) in high-risk pediatric patients remains unclear. Therefore, this study aims to characterize pediatric RSV cases from January 2019 to December 2022 and assess the impact of the COVID-19 pandemic on RSV burden and RSV-related outcomes. In addition, examining factors influencing RSV-related hospitalization.

**Methods:**

This is a retrospective study that included pediatric patients (aged 14 and below) who presented at King Faisal Specialist Hospital and Research Centre (KFSHRC) in Riyadh, Saudi Arabia with RSV infection identified using real-time reverse-transcriptase polymerase chain reaction assays. Statistical analyses were performed using STATA.

**Results:**

A total of 885 RSV cases were reported; (56.05%) were males and (43.95%) were females with a median age of 24 months [interquartile range (IQR): 11–60]. 534 (60.34%) required hospitalization. As for RSV seasonality, there was a significant increase in RSV prevalence following the COVID-19 pandemic, escalating from 205 cases in 2019 to 425 cases in 2022. The increase in 2022 was evident in January and persisted from September to December, reaching its peak during the months of October (20.70% − 88 cases) and November (32.00% − 136 cases). About (27.12%) of RSV infected children were medically free patients. Symptomatic patients exhibited various clinical manifestations, with ventilation necessary in (13.11%) of cases. Further analysis revealed significant changes in RSV-related outcomes post-COVID-19, including a decrease in hospitalization rates, an increase in medically free patients, and a lower need for ventilation (*p* < 0.05). Notably, a significant proportion of RSV admissions occurred within the first 6 months of life, with (77.69%) in the age group of 0 to 5 months. In addition, previous RSV infection, prematurity, low birth weight, renal disease, congenital heart disease, endocrine/metabolic disease, neuro/neuromuscular diseases, and genetic disorders were positively associated with hospitalization (*P* < 0.05). Interestingly, asthma and bone marrow transplantation were negatively associated with hospitalization (*P* < 0.05). The mortality rate in this study is (2.37%) (21/885).

**Conclusion:**

This study provides a comprehensive understanding of the demographic and clinical factors influencing RSV outcomes, highlighting the impact of the COVID-19 pandemic and shedding light on potential risk factors for RSV-related hospitalization. The highest prevalence of RSV during (September to January), aligning with global patterns and emphasizing the importance of timing in preventive strategies.

**Supplementary Information:**

The online version contains supplementary material available at 10.1007/s44197-024-00218-4.

## Introduction

Respiratory syncytial virus (RSV) belongs to the Pneumoviridae family, a single-stranded and negative-sense RNA virus. RSV can be grouped according to two main antigens, A and B subtypes [1]. RSV typically spreads during winter epidemics in temperate climates and rainy seasons in tropical regions [2]. Although shifts in seasonality occurred during the coronavirus disease 2019 (COVID-19) pandemic, with an unusually early surge in (RSV) diseases post-COVID probably as a consequence of lifting COVID-19 precautions [3, 4].

In a recent European study, approximately 50% of hospitalizations for respiratory tract illness in children younger than one year were associated with RSV. Approximately 60% of these illnesses occurred in infants younger than three months of age [5]. In low-income countries, more than 80% of RSV- attributable deaths are estimated to not occur in the hospital [6]. In addition to well-known risks for severe RSV disease, such as prematurity, bronchopulmonary dysplasia, and congenital heart disease [6], it has been shown that patients with other conditions such as immunodeficiency, recipients of hematopoietic stem cell transplant (HSCT) and patients with hematologic malignancy also have high disease severity and mortality risk [7–14].

The currently available antivirals are limited in efficacy and/or associated with potential toxicity, thus emphasizing the importance of prevention strategies [15–17]. Palivizumab, an RSV-specific recombinant monoclonal antibody, or polyclonal IVIG, has been used in combination with Ribavirin. However, their contribution to RSV treatment is controversial, and difficult to quantify their cost [15, 16]. Recently two promising prevention strategies became available for protecting all infants against RSV, including passive immunization of young infants through vaccination of pregnant women (maternal immunization) and administration of long-acting monoclonal antibodies (mAbs) to neonates and infants [18–22]. Despite the recent progress in preventing RSV disease in neonates and infants, developing prevention strategies against RSV for all high-risk infants and children entering their first RSV season still constitutes a significant unmet medical need.

Therefore, this study aims to characterize pediatric RSV cases from January 2019 to December 2022 and assess the impact of the COVID-19 pandemic on RSV epidemiology. This study will help policymakers to plan appropriate prevention strategies.

## Methods

King Faisal Specialist Hospital and Research Centre (KFSHRC) is a tertiary referral hospital that provides inpatient and outpatient general and highly specialized medical treatment, transplantation, and oncology services. This is a retrospective single-center study that included pediatric RSV patients (aged 14 years and below) who presented at KFSHRC in Riyadh, Saudi Arabia from (January 2019 to December 2022) with RSV infection identified by a qualitative real-time reverse-transcriptase polymerase chain reaction assay in nasopharyngeal swab or bronchoscopy specimens. Patients who lacked laboratory confirmation tests were excluded.

Data was gathered by electronic chart review and then stored in REDCap (10.8.0 - © 2021 Vanderbilt University). The extracted data included the following: epidemiology, medical history, clinical manifestations, management, complications, and outcomes. The study was conducted in accordance with STROBE criteria. The study sample were classified into three groups based on periods of the COVID-19 pandemic: pre-COVID-19 (2019), during COVID-19 (2020–2021), and post-COVID (2022).

Analysis was done using STATA software version 18. Categorical variables were reported as frequency (n) and (%). Continuous variables were reported as median and interquartile range [IQR]. In addition, the Chi-square test was done to compare clinical characteristics before and after COVID-19. Also, univariate logistic regression was done to investigate factors related to hospitalization. The significance threshold is set at p-value < 0.05.

## Results

A total of 885 cases were reported; 389 cases (43.95%) were female, while 496 cases (56.05%) were male. The median age of the patients was 24 months [IQR: 11, 60]. Additionally, most patients had underlying conditions including congenital heart disease (164 cases, 18.53%), prematurity (72 cases, 8.14%), and asthma (68 cases, 7.68%). In addition, 68 (7.68%) had hematopoietic stem cell transplant (HSCT) and 71 (8.02%) had solid organ transplant, including liver (48 cases, 67.61%), kidney (17 cases, 23.94%), heart (4 cases, 5.63%), lung (1 case, 1.41%), and small bowel (1 case, 1.41%) (see Table 1).

**Table 1:** Characteristics of reported RSV episodes (*n* = 885)

Characteristics	n (%), Median [IQR]
*Gender*	
-Female	389 (43.95)
-Male	496 (56.05)
*Age (months)*	24 [11–60]
*RSV cases distribution*	
-Pre-COVID-19	205 (23.16)
-During-COVID-19	255 (28.81)
-Post-COVID-19	425 (48.02)
*Previous RSV infection*	46 (5.20)
*Previous use of Palivizumab*	21 (2.37)
*Underlying disease*	
-Prematurity (GA < 37 weeks)	72 (8.14)
-Low birth weight (BW < 2.5 kg)	32 (3.62)
-Hematological malignancies	64 (7.23)
-Hematological diseases (non-oncology)	54 (6.10)
-Immunodeficiency	51 (5.76)
-Solid tumors	24 (2.71)
-Renal disease	48 (5.42)
-Liver diseases	61 (6.89)
-Congenital heart disease	164 (18.53)
-Endocrine/metabolic disease	59 (6.67)
-Neuro/neuromuscular diseases	93 (10.51)
-Asthma	68 (7.68)
-Chronic lung disease	15 (1.69)
-Congenital anomalies	17 (1.92)
-Gastrointestinal	5 (0.56)
-Genetic disorder	27 (3.05)
-Cystic fibrosis	6 (0.68)
-Other	13 (1.47)
-Medically free	240 (27.12)
*Hematopoietic stem cell transplant*	68 (7.68)
*Solid organ transplant*	71 (8.02)
-Liver	48 (67.61)
-Kidney	17 (23.94)
-Heart	4 (5.63)
-Lung	1 (1.41)
-Small bowel	1 (1.41)
*Immunosuppressive therapy*	66 (7.46)
*Steroid therapy*	41 (4.63)
*Chemotherapy*	62 (7.01)

RSV: respiratory syncytial virus, GA: gestational age, BW: birth weight

**Fig. 1:**
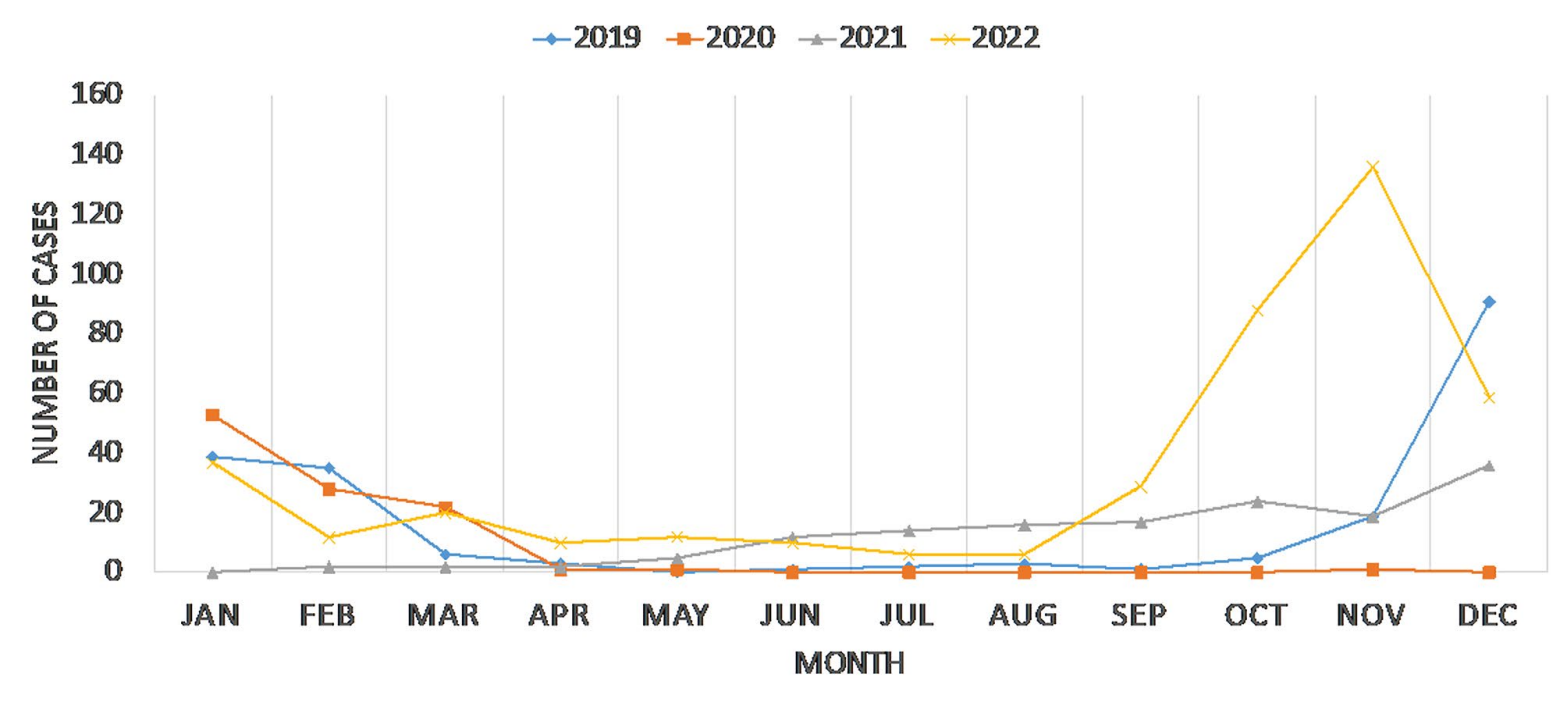
The seasonality of reported Respiratory Syncytial Virus (RSV) cases from January 2019 to December 2022

RSV cases were categorized based on their occurrence in relation to the COVID-19 pandemic, with 205 cases (23.16%) reported as pre-COVID-19, 255 cases (28.81%) during COVID-19, and 425 cases (48.02%) post-COVID-19 (see Table 1). As for seasonality of RSV infection, in 2019, there were 205 reported cases of RSV, peaking in January (19.02% − 39 cases) and December (44.39% − 91 cases). In 2020, 106 cases were reported, with the highest incidence in January (50% − 53 cases). In 2021, there were 149 cases, peaking in December (24.16% − 36 cases). Notably, in 2022, there was a substantial increase with 425 reported cases; the increase in 2022 was evident in January and persisted from September to December, reaching its peak during the months of October (20.70% − 88 cases) and November (32.00% − 136 cases) (See Fig. 1).

**Fig. 2:**
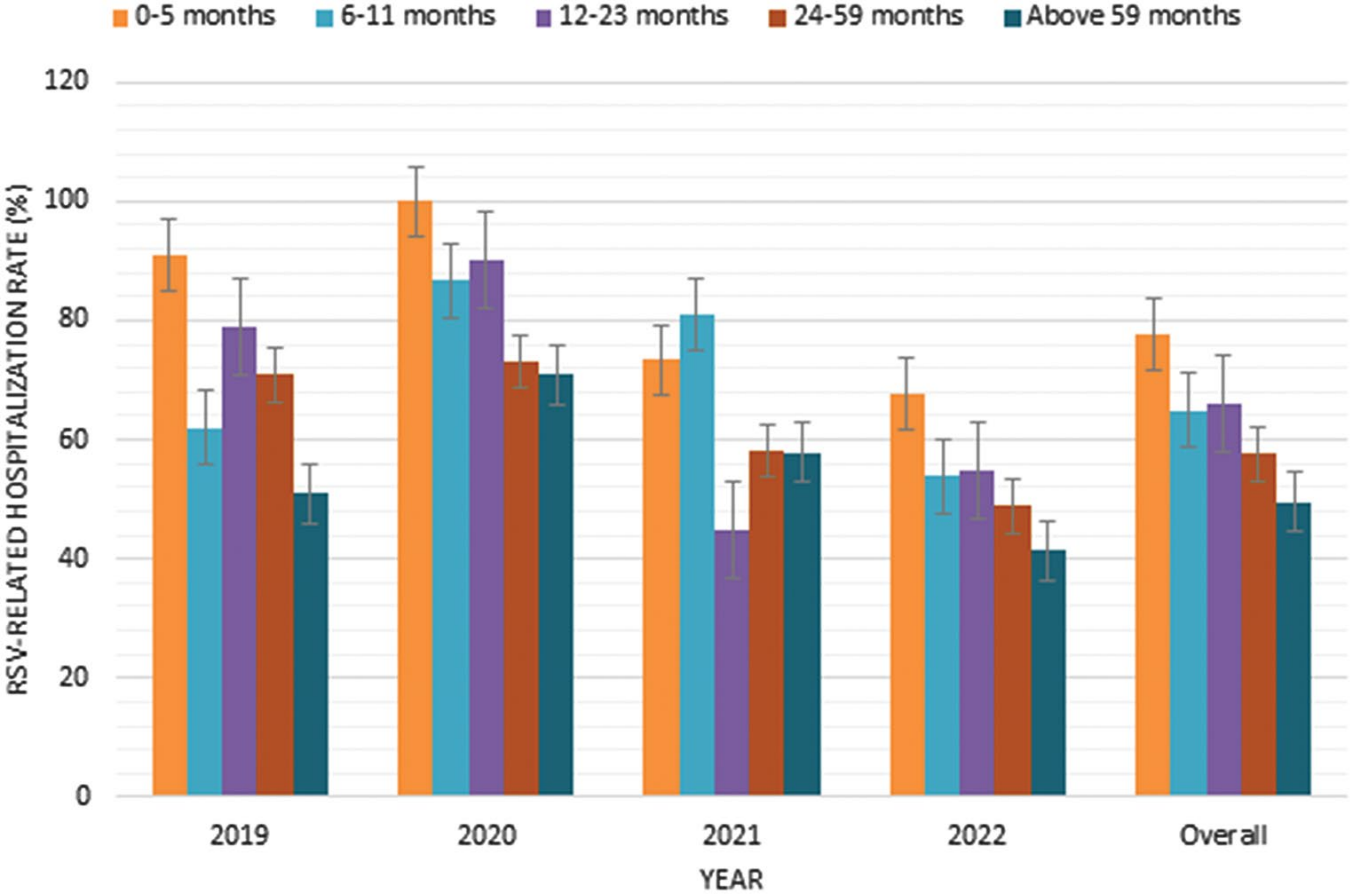
RSV-related hospitalization rates per age group from 2019 to 2022

Notably, (73.71%) of cases required admission during RSV, and 140 patients (26.22%) were admitted to the Intensive Care Unit (ICU) during their illness (see Table 2). When analyzing RSV-related hospitalization rates from 2019 to 2022 across different age groups, a predominant occurrence was observed in the 0–5 months age group in 2019 (90.91%). Subsequent years showed fluctuations, with a notable increase in 2020, particularly in the 0–5 months age group (100%). However, in 2021, there was a decrease in the 12–23 months age group, and in 2022, percentages generally declined across all age groups compared to previous years. This suggests a shifting trend in the distribution of RSV-related hospitalizations over the specified age ranges during the four-year period (see Fig. 2). Overall, a substantial proportion of RSV admissions occurred within the first 6 months of life, constituting 77.69% in the age group of 0 to 5 months.

**Table 2:** Hospital course and outcome of RSV patients

Characteristics	n (%), Median [IQR]
*Required admission during RSV*	534 (60.34)
-General ward	394 (73.78)
-ICU	140 (26.22)
*Presentations*	
-Lethargy	102 (11.53)
-Gastrointestinal symptoms	163 (18.42)
-Respiratory distress	221 (24.97)
-Runny nose	449 (50.73)
-Fever	676 (76.38)
-Cough	716 (80.90)
-Tachycardia	37 (4.18)
-Hypotension	7 (0.79)
-Other	65 (7.34)
-Asymptomatic	18 (2.03)
Ventilation*	116 (13.11)
-Mechanical ventilation	19 (16.38)
-HFNC	80 (68.97)
-Non-invasive ventilation	41 (35.34)
*Viral Co-infection*	284 (32.09)
*Use of antimicrobials*	528 (59.66)
*Outcome*	
-Recovered	851 (96.16)
-Died	21 (2.37)
-Unknown/not documented	13 (1.47)
*Hospital stay (days)*	6 [3–11]
*ICU stay (days) (* *n* * = 140)*	5 [2, 12]
Serious Infection**	73 (8.25)
-Bacteremia	30 (41.10)
-UTI	36 (49.32)
-Complicated pneumonia	15 (20.55)

*22 patients were on more than one type of ventilation

**8 patients had more than one type of serious infection

RSV: respiratory syncytial virus, ICU: intensive care unit, HFNC: high-flow nasal cannula, UTI: urinary tract infection

Symptomatic patients exhibited a range of clinical manifestations, including fever (76.38%), cough (80.90%), runny nose (50.73%), and respiratory distress (24.97%). Ventilation was necessary for 116 cases (13.11%), with 19 patients (16.38%) requiring mechanical ventilation, 80 patients (68.97%) requiring high-flow nasal cannula (HFNC), and 41 patients (35.34%) requiring non-invasive ventilation. Viral co-infection was observed in 32.09% of cases, most commonly Rhinovirus/Enterovirus, followed by Adenovirus and Influenza. In addition, antimicrobials were administered in 59.66% of cases. The overall outcome revealed that most patients recovered (96.16%), while 21 patients (2.37%) succumbed to the infection, and the outcome for 13 cases (1.47%) was unknown or not documented. The median hospital stay was 6 days (IQR: 3–11), and for those admitted to the ICU (*n* = 140), the median ICU stay was 5 days (IQR: 2–12). Serious infections were reported in 73 (8.25%) patients, including bacteremia 30 (41.10%), urinary tract infection (UTI) 36 (49.32%), and complicated pneumonia 15 (20.55%). It is noteworthy that eight patients experienced more than one type of serious infection (See Table 2).

The univariate analysis showed several notable differences post-COVID-19. The proportion of patients requiring hospitalization during RSV significantly decreased from (68.78%) pre-COVID-19 to (50.82%) post-COVID-19 (*p* = 0.000). Other significant changes post-COVID-19 include an increase in medically free patients (22.44–34.59%, *p* = 0.002), and a reduction in the need for ventilation (17.56–10.35%, *p* = 0.011). There was a non-significant increase in in-hospital mortality from (1.95%) pre-COVID-19 to (2.12%) post-COVID-19 (*p* = 0.250) (See Table 3).

**Table 3:** Univariate analysis to compare characteristics pre and post-COVID-19

Characteristics	Pre-COVID-19 (n = 205)	Post-COVID-19 (n = 425)	P-value
*Age less than 6 months*	33 (16.10)	68 (16.00)	0.975
*Hospitalization during RSV*	141 (68.78)	216 (50.82)	0.000*
-Length of stay ≥ 7 days	67 (47.52)	87 (40.09)	0.166
*ICU admission*	36 (17.56)	51 (12.00)	0.058
*Medically free*	46 (22.44)	147 (34.59)	0.002*
*Ventilation*	36 (17.56)	44 (10.35)	0.011*
*Viral co-infection*	68 (33.17)	139 (32.71)	0.907
*Serious infection*	16 (7.80)	29 (6.82)	0.654
*In-hospital mortality*	4 (1.95)	9 (2.12)	0.890

Data were reported as frequency (%), and p-values were reported using the Chi-square test

When examining factors related to RSV related hospitalization, age below 6 months was a significant factor with an odds ratio of 2.58 (95% CI: 1.67–4.01, *p* = 0.000), indicating that patients in this age group are more likely to be hospitalized. Other significant factors associated with increased odds of hospitalization include previous RSV infection (OR: 2.16, 95% CI: 1.08–4.32, *p* = 0.028), prematurity (GA < 37 weeks) (OR: 2.67, 95% CI: 1.49–4.80, *p* = 0.001), low birth weight (BW < 2.5 kg) (OR: 2.94, 95% CI: 1.19–7.22, *p* = 0.018), renal disease (OR: 2.30, 95% CI: 1.15–4.57, *p* = 0.017), congenital heart disease (OR: 2.26, 95% CI: 1.54–3.31, *p* = 0.000), endocrine/metabolic disease (OR: 3.92, 95% CI: 1.90–8.08, *p* = 0.000), neuro/neuromuscular diseases (OR: 2.81, 95% CI: 1.66–4.74, *p* = 0.000), and genetic disorder (OR: 3.90, 95% CI: 1.33–11.38, *p* = 0.013). However, asthma and bone marrow transplantation (BMT) were negatively associated with hospitalization (OR: 0.52, 95% CI: 0.31–0.86, *p* = 0.011), (OR: 0.40, 95% CI: 0.24–0.66, *p* = 0.000), respectively. Finally, around 21 (2.37) patients had previously received Palivizumab with no effect on hospitalization (OR: 0.60, 95% CI: 0.23, 1.56, *p* = 0.298) (See Table 4).

**Table 4:** Factors related to hospitalization

Characteristics	OR [95% CI]	P-value
*Age below 6 months*	2.58 [1.67, 4.01]	0.000*
-Male gender	1.124 [0.94, 1.63]	0.119
*Previous RSV infection*	2.16 [1.08, 4.32]	0.028*
*Palivizumab*	0.60 [0.23, 1.56]	0.298
-Prematurity (GA < 37 weeks)	2.67 [1.49, 4.80]	0.001*
-Low birth weight (BW < 2.5 kg)	2.94 [1.19, 7.22]	0.018*
-Hematological malignancies	1.10 [0.65, 1.86]	0.714
-Hematological diseases (non-oncology)	0.59 [0.34, 1.02]	0.061
-Immunodeficiency	1.01 [0.57, 1.81]	0.947
-Solid tumors	1.61 [0.66, 3.93]	0.291
-Renal disease	2.30 [1.15, 4.57]	0.017*
-Liver diseases	1.76 [0.99, 3.14]	0.054
-Congenital heart disease	2.26 [1.54, 3.31]	0.000*
-Endocrine/metabolic disease	3.92 [1.90, 8.08]	0.000*
-Neuro/neuromuscular diseases	2.81 [1.66, 4.74]	0.000*
-Asthma	0.52 [0.31, 0.86]	0.011*
-Chronic lung disease	2.66 [0.74, 9.51]	0.131
-Congenital anomalies	3.12 [0.89, 10.94]	0.075
-Genetic disorder	3.90 [1.33, 11.38]	0.013*
*HSCT*	0.40 [0.24, 0.66]	0.000*
*Solid organ transplant*	1.51 [0.89, 2.54]	0.121

OR: odds ratio, CI: confidence interval, P-values were reported using univariate logistic regression

## Discussion

In this research, we described the epidemiological patterns of RSV infection among pediatric patients at a tertiary care center in Saudi Arabia before, during, and after the COVID-19 pandemic. The burden of RSV-related hospitalizations in the pediatric population in Saudi Arabia was substantial, accounting for (60.34%) of cases. Notably, a significant proportion of RSV admissions occurred within the first 6 months of life, with (77.69%) in the age group of 0 to 5 months. This is consistent with published data as a study by Alkharsah (2022) conducted in a tertiary care hospital in the Eastern Province of Saudi Arabia reported that hospitalization rates were notably elevated in infants and younger children, showing a significant decrease with increasing age (p-value < 0.001) [23]. Similarly, a study conducted in Canada by Bourdeau et al. (2023) found that a significant proportion of RSV admissions occurred within the first 6 months of life, with nearly (40%) in the age group of 0 to 2 months [24].

Our current study revealed that only (27.12%) of RSV-infected patients were medically free. However, it’s worth noting that this figure may be underestimated due to our status as a large referral center with specialized services in immunology and oncology, leading to a patient population predominantly with pre-existing medical conditions. Consequently, the number of medically free patients encountered may not be generalizable. Nonetheless, this proportion remains significant, considering that these individuals occupy beds crucially needed for other patients with complex conditions. On the contrary, another study conducted in the Eastern province of Saudi Arabia by AlBahrani et al. (2024) reported that (62.7%) of RSV-infected patients did not have other comorbidities [25]. This is similar to another reported study in Norway by Havdal et al. (2021), as they reported (85%) RSV-infection in patients with no pre-existing medical conditions [26].

As for RSV seasonality, there was a significant upswing in RSV prevalence following the COVID-19 pandemic, escalating from 205 cases in 2019 to 425 cases in 2022, likely due to pandemic restrictions being lifted. The increase in 2022 was evident in January and persisted from September to December, reaching its peak during the months of October and November. Similarly, Alkharsah (2022) reported that prevalence of RSV infection exhibited a higher incidence from August to February, experiencing a substantial decline from March to July. The pinnacle of infection occurred during the months of December and January [23]. Additionally, according to AlBahrani et al. (2024), RSV cases increased from (42 in 2019 to 78 cases in 2022), the greatest number of cases occurred in December 2019 (29, 19.3%), October 2022 (29, 19.3%), and November 2022 (25, 16.7%) [25].

This study shows that the RSV burden also includes healthy or medically free patients, as evident by the increased number of post COVID-19. In addition, literature on this topic shows that RSV infection occurs in medically free children. This emphasizes the need for preventive strategies that also target non-high-risk individuals.

The Centers for Disease Control (CDC) have made recommendations regarding to the seasonality of administering preventive measures for RSV. In addition, they have recommended Nirsevimab to be given to all infants younger than age 8 months who are born shortly before or during their first RSV season (typically fall through spring) who meet the required criteria [27]. Nirsevimab, a monoclonal antibody designed to target the prefusion conformation of the RSV F glycoprotein, exhibits a prolonged half-life and robust neutralizing capabilities. A single dose of Nirsevimab is anticipated to offer protection for a minimum of five months, simplifying the vaccination process to just one dose at the commencement of the RSV season. The US Food and Drug Administration granted approval for Nirsevimab’s use in all infants in 2023. Notably, it is recommended as a replacement for palivizumab, even in premature infants and those with additional risk factors, unless Nirsevimab is unavailable [28, 29].

However, it is important to note that other vaccines such as Pfizer Abrysvo and GSK Arexvy RSV are not recommended for use in young children [27], because their safety and effectiveness have not yet been established when administered to infants and children under age 2 years [30].

## Limitations

Overall, the study provides a detailed snapshot of the demographic and clinical characteristics of RSV episodes, and the inclusion of a significant sample size (885 cases) further enhances the study’s robustness, aiding in understanding its epidemiology and evolution after the COVID-19 pandemic. However, certain limitations should be considered. The retrospective nature of the study and its single-center design may introduce inherent biases, potentially limiting the generalizability of the findings.

## Conclusion

In conclusion, this comprehensive study provides a nuanced understanding of pediatric RSV infections over a four-year period, shedding light on the impact of the COVID-19 pandemic on RSV epidemiology. The burden of RSV-related hospitalizations, particularly in infants under six months, underscores the vulnerability of this age group to severe outcomes, in addition to previously healthy infants. The highest prevalence of RSV during (September to January), aligning with global patterns and emphasizing the importance of timing in preventive strategies.

Despite recent progress in preventive strategies, challenges persist in addressing the needs of high-risk infants entering their first RSV season. The study provides a solid foundation for ongoing research and the development of tailored prevention strategies. Overall, this research contributes substantially to the existing knowledge on pediatric RSV infections, guiding public health initiatives and clinical interventions for improved outcomes.

### Electronic Supplementary Material

Below is the link to the electronic supplementary material

Supplementary Material 1

## Data Availability

No datasets were generated or analysed during the current study.
