# Supplementary material for: Evolving Epidemiology of Pediatric Respiratory Syncytial Virus (RSV) Cases Around COVID-19 Pandemic: Impact and Clinical Insights, Retrospective Cohort Study
Source: J Epidemiol Glob Health. 2024 Apr 4;14(2):319–26. doi: 10.1007/s44197-024-00218-4 (PMC11176150; doi:10.1007/s44197-024-00218-4)
Supplement: Supplementary file 1 — Supplementary Material 1 [file 44197_2024_218_MOESM1_ESM.docx]

**Supplementary table:**

**S.1: Univariate analysis to compare characteristics between medically free patients versus non-medically free (n = 885):**

| **Characteristics** | **Medically free patients (n = 240)** | **Non-medically free patients (n = 645)** | **P-value** |
| --- | --- | --- | --- |
| ***Age less than 6 months*** | 72 (30.00) | 58 (8.99) | 0.000* |
| ***Previous RSV infection*** | 7 (2.92) | 39 (6.05) | 0.062 |
| ***Hospitalization during RSV*** | 94 (39.17) | 440 (68.22) | 0.000* |
| -Length of stay ≥ 7 days | 22 (23.40) | 221 (50.23) | 0.000* |
| ***ICU admission*** | 22 (9.17) | 118 (18.29) | 0.001* |
| ***Ventilation*** | 25 (10.42) | 91 (14.11) | 0.148 |
| ***Viral co-infection*** | 84 (35.00) | 200 (31.01) | 0.258 |
| ***Serious infection*** | 8 (3.33) | 65 (10.08) | 0.001* |
| ***In-hospital mortality*** | 1 (0.42) | 20 (3.10) | 0.020* |

Data were reported as frequency (%), and p-values were reported using the Chi-square test.

**S.2: Sub-groups analysis to compare characteristics pre and post-COVID-19 in medically free versus non-medically free patients:**

| **Characteristics** | **Medically free patients (n = 193)** | | | **Non-medically free patients (n = 437)** | | |
| --- | --- | --- | --- | --- | --- | --- |
|  | **Pre-COVID-19**  **(n = 59)** | **Post-COVID-19**  **(n = 121)** | **P-value** | **Pre-COVID-19**  **(n = 159)** | **Post-COVID-19**  **(n = 278)** | **P-value** |
| ***Age less than 6 months*** | 22 (47.83) | 35 (23.81) | 0.002* | 11 (6.92) | 33 (11.87) | 0.098 |
| ***Hospitalization during RSV*** | 30 (65.22) | 44 (29.93) | 0.000* | 111 (69.81) | 172 (61.87) | 0.095 |
| -Length of stay ≥ 7 days | 11 (36.67) | 5 (11.36) | 0.009* | 56 (50.45) | 82 (47.40) | 0.616 |
| ***ICU admission*** | 10 (21.74) | 5 (3.40) | 0.000* | 26 (16.35) | 46 (16.55) | 0.958 |
| ***Ventilation*** | 12 (26.09) | 7 (4.76) | 0.000* | 24 (15.09) | 37 (13.31) | 0.604 |
| ***Viral co-infection*** | 10 (21.74) | 61 (41.50) | 0.015* | 58 (36.48) | 78 (28.06) | 0.067 |
| ***Serious infection*** | 3 (6.52) | 3 (2.04) | 0.126 | 13 (8.18) | 26 (9.35) | 0.678 |
| ***In-hospital mortality*** | 0 (0.00) | 0 (0.00) | NA | 4 (2.52) | 9 (3.24) | 0.669 |

Data were reported as frequency (%), and p-values were reported using the Chi-square test.
